# Contamination of Public Buses with MRSA in Lisbon, Portugal: A Possible Transmission Route of Major MRSA Clones within the Community

**DOI:** 10.1371/journal.pone.0077812

**Published:** 2013-11-06

**Authors:** Teresa Conceição, Fernanda Diamantino, Céline Coelho, Hermínia de Lencastre, Marta Aires-de-Sousa

**Affiliations:** 1 Laboratory of Molecular Genetics, Instituto de Tecnologia Química e Biológica (ITQB), Universidade Nova de Lisboa (UNL), Oeiras, Portugal; 2 Faculdade de Ciências e Centro de Estatística e Aplicações, Universidade de Lisboa (FCUL e CEAUL), Lisboa, Portugal; 3 Laboratory of Microbiology, The Rockefeller University, New York, New York, United States of America; 4 Escola Superior de Saúde da Cruz Vermelha Portuguesa (ESSCVP), Lisboa, Portugal; Columbia University, United States of America

## Abstract

In a previous study we have shown that public buses in Oporto, the second largest city in Portugal, were highly contaminated with MRSA. Here we describe the results of a similar study performed in another urban area of Portugal–Lisbon, the capital. Between May 2011 and May 2012, hand touched surfaces of 199 public buses in Lisbon were screened for MRSA contamination. Subsequently, the hands of 575 passengers who frequently use these bus lines were also screened. All hand carriers of MRSA were further screened for nasal carriage. The isolates were characterized by PFGE, staphylococcal cassette chromosome (SCC) *mec* typing, *spa* typing, MLST and were tested for the presence of *mecA*, Panton-Valentine leukocidin and arginine catabolic mobile element genes. MRSA contamination was shown in 72 buses (36.2%). The majority of the isolates belonged to three major clones: Clone A was identified as EMRSA-15 defined by pattern PFGE A, *spa* types t2357/t747/t025/t379/t910, ST22, and SCC*mec* IVh (n = 21; 29%). Clone B was the New York/Japan clone characterized by PFGE B-t002/t10682-ST5-II (n = 15; 21%). Clone C included isolates with characteristics of the international community-acquired USA300 or related clones, PFGE C-t008-ST8-IVa/IVc/IVg/IVnt/VI (n = 19; 26%). The first two clones are currently the two major lineages circulating in Portuguese hospitals. The hands of 15 individuals were contaminated with MRSA belonging to the nosocomial clones A or B. Eleven of these individuals were not nasal carriers of MRSA and all but one had travelled by public transportation, namely by bus, prior to sampling. In conclusion, public buses in two major cities in Portugal are often contaminated with MRSA representing clones dominant in hospitals in the particular geographic area. MRSA contamination of public transport and the transfer of the bacteria to the hands of passengers may represent a route through which hospital-acquired MRSA clones may spread to the community.

## Introduction

Methicillin-resistant *Staphylococcus aureus* (MRSA) is a major healthcare associated (HA-MRSA) human pathogen responsible for mild to severe life threatening infections worldwide [1], [2]. Since the mid-1990 s, MRSA has also been identified as the etiological agent of infections acquired in the community (CA-MRSA) [3]–[5]. A major concern for public health emerged with the blurring of the molecular and epidemic boundaries between HA- and CA-MRSA: CA-MRSA strains originally confined to the community are increasingly causing healthcare-acquired infections as indicated by the rise in the proportion of USA300 isolates causing invasive infections in United States hospitals [6], [7].

Although, in 2011 the EARSS-Net reported stabilizing or even decreasing MRSA rates in several European countries, Portugal continues to show an increasing trend, reaching a nosocomial prevalence of 54.3%, which represents the highest rate in Europe [2]. A few highly disseminated clones are currently responsible for HA-MRSA infections in the country, namely EMRSA-15 (ST22-IV) – which has been endemic in Portuguese hospitals since 2001 - and the New York/Japan clone (ST5-II), which is currently the second most prevalent lineage [8]–[11].

In contrast, until very recently, the MRSA prevalence in the community has been extremely low. Studies including isolates from nasal swabs of young healthy individuals, nasopharyngeal swabs of children attending day care centers and isolates from soft tissues infections (SSTI) in children attending a pediatric emergency department, reported a prevalence of MRSA lower than 0.9% [12]–[14]. Typically CA-MRSA clonal lineages, as the European clone (ST80-IVc) or the USA300 clone (ST8-IVa) producing PVL, were sporadically reported in Portugal only in cases where the existence of hospital risk factors could not be eliminated [12], [15], [16].

However, in 2010, the MRSA rate reached 25.9% among SSTI in adults attending heath care centers [11] and in 2013 a very high prevalence of CA-MRSA (21.6%) was reported among individuals with no risk factors who attended healthcare institutions in Portugal [17]. In both cases most of the isolates belonged to the major HA-MRSA clones. Moreover, a recent study showed that public buses circulating in Oporto, the second largest city of Portugal, were frequently contaminated with MRSA strains which belonged to the HA-MRSA clone, EMRSA-15, a clone which until recently seemed to be strictly confined to hospitals. The detection of this clone on hand-held surfaces of public buses is an ominous sign suggesting a route through which nosocomial MRSA clones may be escaping to the community environment [18].

The purpose of the present study was to extend these tests to public buses circulating in another urban area in the country - Lisbon, the capital of Portugal. Since *S. aureus* transmission occurs mainly by skin contact, we also screened the hands of individuals who frequently travelled by bus and who may represent a stage in the spread of MRSA clones within the community.

## Materials and Methods

### Ethics Statement

The study and the protocol have been previously presented to all students of Escola Superior de Saúde da Cruz Vermelha Portuguesa (ESSCVP) and a verbal informed consent was obtained for each participant (all aged over 18) at the time of hand and nasal screenings, which was registered in the respective record sheet.

The study and the consent procedure were approved by the Research Department and Ethics Committee of ESSCVP (Gabinete de Investigação and Centro de Bioética da ESSCVP).

### Sampling of Vehicles

The transportation agency to which the buses belong is a major public transportation network in Lisbon, with 78 different routes served by 735 vehicles. Daily cleaning (at the end of the day) of the interior of the vehicles is limited to trash removal and floor vacuuming, while in depth cleaning, which occurs every three months only, includes cleaning of the hand touched surfaces with an alkaline detergent (VQ 300 Desengordurante Multi Usos).

In sampling for MRSA contamination, we selected surfaces with high levels of hand contact. These included handrails, seat rails, handgrips, stop buttons and the surface of the validation tickets machine. Sampling was always conducted in both sides of a bus, and an attempt was made to sample the same number of surfaces per bus. Sampling was performed every two or three weeks between May 2011 and May 2012 (with the exception of August), at the end of the day and before any cleaning procedure took place.

An attempt was made to collect samples from a variety of routes. In each sampling day, six to 12 buses were arbitrarily chosen, some serving major hospitals and others not. Since vehicles were usually assigned to a different route each day, the vehicle identifier number and the route of service were registered at the time of sampling.

Samples were collected using two sterile cotton gauzes per vehicle (one for each side), moistened with Tryptic Soy Broth (TSB) (Becton, Dickinson & Co, New Jersey, USA). After swabbing the hand touched surfaces, the gauzes were introduced into 100 ml TSB bottles and taken to the laboratory for incubation.

### Hand Screening

Samples were recovered in two periods: in May 2012 for 2 days and in January 2013 for 3 consecutive days. The hands of students and workers who travelled by bus or by other means of public transportation to ESSCVP in Lisbon were swabbed. Individuals were screened in the morning, between 8.00 am and 10.00 am, immediately after entering the school building and before they could wash their hands or touch other surfaces. Samples were collected by swabbing both hands of each individual with a sterile cotton gauze moistened with sterile water and then submerged into TSB for further transport to the laboratory.

Individuals showing MRSA contamination on their hands were further swabbed for nasal carriage within a week in order to identify MRSA nasal colonization. Whenever possible, a second hands screening was performed at the same time to reevaluate the persistence or re-contamination of the hands. Nasal screenings were performed on both nares of each individual with a sterile dry cotton swab, which was stored in Stuart transport medium and transferred to the laboratory.

### Bacterial Isolation

All gauzes, from buses and hand screenings, were incubated in TSB at 37°C overnight with orbital agitation (135 rpm) for enrichment and then plated with a sterile cotton swab, onto Tryptic Soy Agar (TSA) (Becton, Dickinson & Co) and onto a chromogenic selective media for MRSA, CHROMagar MRSA (CHROMagar, Paris, France). Nasal swabs were directly plated onto TSA and CHROMagar MRSA. After 24 to 48 h of incubation at 37°C, all presumptive MRSA colonies (mauve-colored colonies) were tested for coagulase by latex agglutination test Staphaurex Plus (Remel – Oxoid, Madrid, Spain) or by agglutination of rabbit plasma in tubes (Becton Dickinson & Co, New Jersey, USA) in case of prior ambiguous results.

MRSA isolates were confirmed at the species level and for methicillin resistance by amplification of the *nuc* and *mecA* genes, respectively [19], [20].

### Pulsed-field gel Electrophoresis (PFGE)

PFGE was performed for all isolates after SmaI digestion as described by Chung et al. [21]. The resulting band profiles were analyzed by both visual inspection using the criteria of McDougal et al. [22], followed by automated analysis with the BioNumerics software version 6.6 (Applied Maths, Sint-Martens-Latem, Belgium) for relatedness evaluation to define clusters. Dendrograms were generated as previously described [23] with a lower tolerance value of 1% for band pattern comparisons. Dice coefficient similarity cutoff at 80% and 95% were used for PFGE type and subtype clusters definition, respectively.

### *spa* typing and Multilocus Sequence Typing (MLST)

*spa* typing was performed on at least one representative of each PFGE subtype, as previously described [24], and *spa* types were assigned through the Ridom web server (http://spaserver.ridom.de). Representatives of the different PFGE types were further characterized by MLST [25], [26]. Allelic profiles and sequence types (ST) were defined using the MLST online database (http://www.mlst.net).

### SCC*mec* Typing

Characterization of the staphylococcal cassette chromosome *mec* (SCC*mec*) was performed using specific primers for each SCC*mec* type through a multiplex PCR strategy [27]. For isolates that were non-typeable by this method, the *mec* gene complex class and *ccrAB*/*ccrC* allotypes were determined as previously described [19], [28], [29]. SCC*mec* type IV isolates were subtyped by multiplex PCR [30].

### Detection of Panton-Valentine Leukocidin (PVL) and Arginine Catabolic Mobile Element (ACME)

The presence of PVL was tested in all isolates, as previously described [31]. ACME allotype (I to III) was determined by PCR using primers specific for *arcA* and *opp3* loci [32], [33].

### Statistical Analysis

Bivariate analysis of data was performed using the Statistical Package for Social Science version 19 software (IBM, USA). Chi-square tests were used for comparison of categorical data. Statistical significance was evaluated at α = 0.05.

## Results and Discussion

### MRSA Prevalence in Public Buses

Seventy-two out of the 199 (36.2%) buses that were sampled in Lisbon were contaminated with MRSA. Several factors are expected to contribute to *S. aureus* transmission in vehicles, namely crowdedness, significant hand-to-surface contact, particularly as vehicles become full with passengers, and the fact that there is no possibility for hand hygiene during and immediately after the use of the transport. In a previous study conducted in 2011 in Oporto, the second major city of Portugal (approximately 300 km north of Lisbon), we were able to isolate MRSA from 26% of the sampled buses that belonged to a different transportation agency [18]. Therefore, independently of the transportation agency and geographic area public buses may represent a reservoir of MRSA clones in two major cities in Portugal.

### Sampling Method

Prior studies in other European countries have failed to isolate MRSA from public transport vehicles [34], [35]. In contrast, the two studies performed in public buses in Portugal found high rates of MRSA, which might be due to technical differences in the sampling and culture strategies. The use of a single moistened gauze for the sampling of multiple surfaces instead of dipslides [35] or swabs [34], as well as a pre-enrichment step using a nonselective broth to detect low concentration contamination, may have contributed to the recovery of a significant number of MRSA isolates in the Portuguese studies. However, the technique did not allow determining how many surfaces were positive or the level of contamination of the different surface areas.

### Seasonal Variation

In the present study, contamination of buses did not vary significantly (χ^2^ = 5.169; p = 0.16) according to the sampling season, being 35% (21/60) in the spring, 29% (9/31) in the summer, 50% (24/48) in the fall and 32% (19/60) in the winter. Four months showed a contamination higher than the 36.2% observed for the total study: these were April (42%), October (67%), November (40%) and December (50%). Little is known about seasonal trends in the transmission of *S. aureus* within the community, but several authors have demonstrated seasonal variation in *S. aureus* infections, particularly skin infections, with a preponderance during the summer and fall [36]–[38]. Since we did not screen any bus during the whole month of August, we cannot discard the possibility of a higher contamination in late summer as described for infection.

### Clonal Types of the Isolates Recovered in Portuguese Public Buses

Although the molecular characterization clustered the 72 MRSA isolates recovered into nine clonal types, the overwhelming majority (n = 55; 76%) of the isolates belonged to three main clones (Table 1).

**Table 1:** Molecular characterization of 72 MRSA isolates from public buses and 21 MRSA isolates from human carriage (hands and nasal).

Source	PFGE	*spa* type	ST	SCC*mec*	No isolates	Clonal type	HA/CA-MRSA	No isolates
BUSES	A	t2357/t747/t025/t379/t910	22	IVh	21	EMRSA-15	HA-	21 (29%)
	B	t002/t10682	5	II	15	New York/Japan	HA-	15 (21%)
		t002/t105	5	IVa	3	Pediatric or related	HA-	7 (12%)
		t002/t214/t535		IVc	4			
		t045/t179	5	V	2	Minor	CA-	2
		t010	5	I	1	Minor	HA-	1
	C	t008	8	VI	13	USA300 or related	CA-	19 (26%)
				IVa	4			
				IVc	1			
				IVg	1			
				IVnt	1			
	D	t068	8	IVc	1	USA300 related	CA-	1
	E	t127	1	IVa	1	USA400	CA-	1
	F	t2429	45	V	1	Berlin	HA-	1
	G	t148	72	IVa	1	USA700	CA-	2
				IVc	1			
	H	t002	2628	IVc	1	Pediatric or related	HA-	1
	J	t786	88	IVa	1	Minor	CA-	1
HANDS (1st screening)	A	t032/t2357	22	IVh	10	EMRSA-15	HA-	10
	B	t002	5	II	2	New York/Japan	HA-	2
		t002		IVa	1	Pediatric or related	HA-	3
		t179		IVnt	1			
		t002		IVc	1			
HANDS (2nd screening)	A		22	IVh	2	EMRSA-15	HA-	2
NASAL	A		22	IVh	3	EMRSA-15	HA-	3
	B		5	IVnt	1	Pediatric or related	HA-	1

HA – Healthcare associated; CA – Community-associated.

ST – Sequence type.

nt – non-typeable.

Clone ST22-IVh, the major clone found in the buses in Lisbon, corresponds to the international clone EMRSA-15, which accounted for 54% of the 629 MRSA isolates recovered in 2006 from 11 Portuguese hospitals scattered all over the country and therefore represented the predominant national nosocomial clone at that time [8]. A recent work, including isolates recovered in 2010 in a 1,300-bed tertiary teaching hospital in Lisbon, demonstrated that ST22-IVh has remained the major (72%) nosocomial clone in Lisbon [11]. EMRSA-15 has been found among nasal carriage of healthy dogs in Portugal and may be transmitted to humans in the community [39].

The second most prevalent clone in buses corresponds to the epidemic CA-MRSA clone USA300 and related clones (ST8-IV). A recent study on the population structure of CA-MRSA in Europe showed that most (40%) of the isolates were related to USA300 [16]. USA-300 clone carries ACME and SCC*mec* IVa, which seem to enhance growth and survival of the clone and therefore make it well adapted to the community environment [33]. However, in the present study, only three isolates exhibited the typical characteristics of this clone (ST8-IVa, t008, PVL+, ACME-I). Fourteen ST8 isolates carry a distinct SCC*mec* type (Table 1). These variations may represent a continuous evolution of this clone as suggested by others [40].

The third most prevalent clone in public buses in Lisbon, ST5-II, displays the characteristics of the New York/Japan clone which in 2006 was identified as the major clone in four hospitals located in Lisbon [8]. Among isolates recovered in 2010 from a teaching hospital in Lisbon, the New York/Japan clone or other closely related clones were the second most frequent MRSA lineage (18%) [11].

A most important aspect of our findings is the coincidence between the major clones present in hospitals and the same clones recovered from public buses circulating in the same area. Thus, the two more prevalent nosocomial clones, found in public buses, in the Lisbon study correspond to the main clonal types found in Lisbon hospitals in 2006 [8] and 2010 [11]. On the other hand, 91% of the isolates previously detected in public buses in Oporto belonged to EMRSA-15 [18] which was the major clone in hospitals in the northern region of Portugal [8]. We interpret these findings as indicating that major HA-MRSA clones in Portugal are escaping from the hospitals to the community environment. This conclusion is also in agreement with Tavares et al. who reported for the first time a high prevalence (22%) of CA-MRSA in Portugal with most of the strains belonging to EMRSA-15 [17].

### Hospital/Non-hospital Bus Route

The participating buses were assigned to 33 different routes, out of which seven do not pass near any hospital and the remaining 26 pass at a maximum distance of 200 m of the entrance of at least one hospital: a single hospital (n = 8), two hospitals (n = 6), three hospitals (n = 4), four hospitals (n = 3), five hospitals (n = 3), six hospitals (n = 1) and seven hospitals (n = 1). A total of eight bus routes served more than three hospitals and 14 bus routes presented MRSA contamination superior or equal to the overall prevalence of 36.2% found in this study, showing an association between the proportion of MRSA contamination and a bus route serving at more than three hospitals (χ^2^ = 4.537; p = 0.033).

In a country with a prevalence of nosocomial MRSA over 50% the hands of health care workers, discharged patients, outpatients and hospital visitors leaving the health care institutions and travelling by bus are the most probable transmission route of HA-MRSA to the buses.

### Vehicle Contamination Over Time

**Figure 1:**
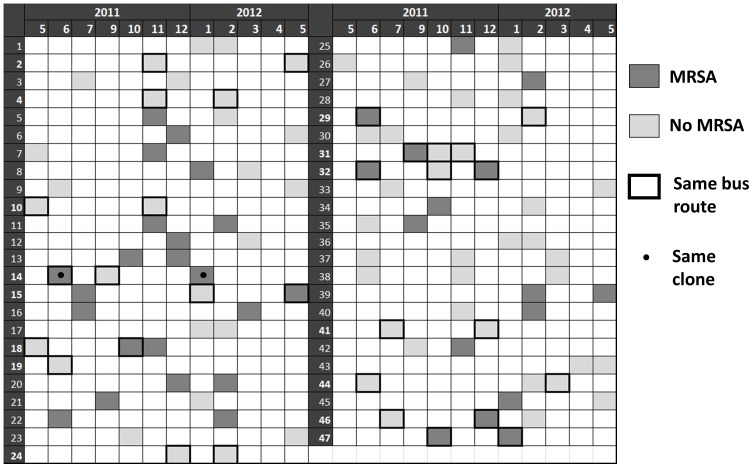
Contamination of vehicles sampled more than once during the study period. Horizontal numbers on the top of the figure represent de year and month of sampling. Vertical numbers correspond to the 47 vehicles identification.

A total of 47 vehicles were screened more than once (Figure 1): two (n = 37) or three times (n = 10). Nineteen of the vehicles were not contaminated with MRSA in any of the screenings, while 10 vehicles were contaminated in at least two screenings. However, from these, only one vehicle showed the same MRSA clone in both occasions, which indicates that MRSA isolates are probably repeatedly inoculated on the hand-touched surfaces of the buses. Although 14 vehicles served the same route in two consecutive screenings, half of them did not maintain the contamination status. Since the screenings were (with a single exception) separated by at least two months, we cannot discard the possibility that in the meantime the vehicles had undergone in depth cleaning.

### Hand Contamination

Among 575 screenings of hands of students and professionals at ESSCVP, 15 individuals presented MRSA contamination, and of these, at least four were found to be MRSA nasal carriers. Eventually, moistened swabs as well as a pre-enrichment step could have improved the sensitivity of the nasal screening. Since the pairs of isolates (hands and nasal) were identical, we assumed that the four individuals were most probably self-contaminated. Moreover, the four individuals were students in clinical training in hospitals and therefore, as health care workers, they have a high risk of MRSA nasal colonization. We considered that 11 out of 575 individuals (2%) presented MRSA contamination on their hands. Molecular characterization distributed the 11 MRSA into three clonal types (Table 1), corresponding to some of the most frequent MRSA clones present in the public buses circulating in Lisbon and all HA-MRSA. Since in the morning of the screening 10 of the 11 individuals travelled to ESSCVP by public transportation, and in all but one case by a bus from the transportation agency that was covered in the present study, it is possible that MRSA contaminating public buses were spread to the hands of its passengers. However, we cannot rule out the possibility that these 11 individuals were colonized at other body sites or got their hands contaminated elsewhere. Future studies sampling the individuals immediately prior and after taking public transportation would be more conclusive.

Five of the 15 individuals showing MRSA on their hands were screened a second time within a week. Two of them showed once again MRSA contamination: (i) one of these individuals was a MRSA nasal carrier and the isolate was identical to the ones previously recovered from the nose and hands; (ii) the second individual was not a nasal MRSA carrier, travelled by public transportation on that day, and showed a different strain relatively to the isolate of the first screening (PFGE A1 instead of A10). These results seem to indicate that MRSA nasal carriage leads to frequent self-contamination of the hands. Moreover, contamination of the hands in non-nasal carriers seems to be transient and probably dependent on the daily public bus transportation.

In summary, public buses in major cities in Portugal (Lisbon and Oporto) are a reservoir of HA- and CA-MRSA clones and may represent a mechanism for the spread of MRSA clones in the community. Given the importance of public transportation in most major cities, the results presented here should provide valuable insights to epidemiologists, infection control and environmental health professionals, to better understand the dynamics of MRSA in the community. The study should also serve as incentive to transportation agencies to introduce more effective procedures to reduce MRSA contamination.
